# Allosteric regulation of kinase activity in living cells

**DOI:** 10.7554/eLife.90574

**Published:** 2023-11-09

**Authors:** Shivani Sujay Godbole, Nikolay V Dokholyan

**Affiliations:** 1 Department of Pharmacology, Penn State College of Medicine Hershey United States; 2 Department of Biomedical Engineering, Penn State University, University Park Hershey United States; 3 Department of Engineering Science and Mechanics, Penn State University, University Park Hershey United States; 4 Department of Biochemistry & Molecular Biology, Penn State College of Medicine Hershey United States; 5 Department of Chemistry, Penn State University, University Park Hershey United States; Goethe University Germany; Goethe University Germany

**Keywords:** protein kinase, allostery, molecular dynamics simulations, protein engineering, None

## Abstract

The dysregulation of protein kinases is associated with multiple diseases due to the kinases' involvement in a variety of cell signaling pathways. Manipulating protein kinase function, by controlling the active site, is a promising therapeutic and investigative strategy to mitigate and study diseases. Kinase active sites share structural similarities, making it difficult to specifically target one kinase, and allosteric control allows specific regulation and study of kinase function without directly targeting the active site. Allosteric sites are distal to the active site but coupled via a dynamic network of inter-atomic interactions between residues in the protein. Establishing an allosteric control over a kinase requires understanding the allosteric wiring of the protein. Computational techniques offer effective and inexpensive mapping of the allosteric sites on a protein. Here, we discuss the methods to map and regulate allosteric communications in proteins, and strategies to establish control over kinase functions in live cells and organisms. Protein molecules, or ‘sensors,’ are engineered to function as tools to control allosteric activity of the protein as these sensors have high spatiotemporal resolution and help in understanding cell phenotypes after immediate activation or inactivation of a kinase. Traditional methods used to study protein functions, such as knockout, knockdown, or mutation, cannot offer a sufficiently high spatiotemporal resolution. We discuss the modern repertoire of tools to regulate protein kinases as we enter a new era in deciphering cellular signaling and developing novel approaches to treat diseases associated with signal dysregulation.

## Introduction

Protein kinases expedite the cell signaling processes by facilitating the transfer of a phosphate from the nucleotide triphosphate to the target protein. Kinase-mediated phosphorylation is critical for a wide spectrum of cell signaling processes such as transcription, translation, cell proliferation, receptor downregulation, metabolism, and apoptosis (Cheng et al., 2011). The dysregulation of kinases alters cell function and may lead to diseases such as schizophrenia (Bentea et al., 2019), Alzheimer’s disease (Ohno, 2014), cancer (Sarkar et al., 2023), and cardiovascular diseases (Chen et al., 2022a). Kinases undergo dynamic conformational changes to elicit their function during cell signaling (Dixon et al., 2004). All kinases have a conserved residue sequence that forms the nucleotide-binding domain to bind to adenosine triphosphate (ATP) or guanosine triphosphate (GTP) (Cheng et al., 2011; Taylor and Kornev, 2011). The binding of the nucleotides is facilitated by phosphorylation of the activation loop (Adams, 2003). Therapies targeting kinases focus on the nucleotide-binding sites (active/orthosteric sites) that regulate the kinase function. Therapies targeting orthosteric sites to regulate kinase activity compete with the native ligand (ATP or GTP) to bind to the active site and need to be highly selective for the active site. Due to the conservation of the nucleotide-binding domain across all protein kinases, specifically targeting the active/orthosteric sites of one type of kinase is challenging.

Kinase activity is also regulated by autoinhibitory domains (Soderling, 1990; Li et al., 2021). Autoinhibitory domains suppress the interaction between the kinases and the effector molecule (nucleotides). The autoinhibitory domains are the sites distal to the active sites that affect the activity of the kinase at the active site through the induction of conformational and dynamic changes in the protein structure (Nussinov and Tsai, 2013; Cui and Karplus, 2008; Guo and Zhou, 2016). Allosteric regulation does not involve direct competition with the native ligand. Instead, it perturbs active site structure or dynamics. The kinases, especially in diseased conditions, may develop resistance to orthosterically regulated therapies by incorporating mutations at the active site, thus enabling kinases to elicit catalytic activity while disallowing the binding of the orthosteric regulators. Allosteric control of kinase function allows the bypass of this resistance (Cui and Karplus, 2008).

Finding allosteric sites that effectively regulate active sites is critical for establishing allosteric control over a particular kinase. X-ray crystallography (Keedy, 2019) and nuclear magnetic resonance (NMR) spectroscopy (Grutsch et al., 2016) techniques are widely used to observe the structures of a protein. The changes occurring in the structure of protein molecules during the allosteric binding of effector molecules can be mapped by comparing structures at different points in time. However, these techniques are expensive and time-consuming. Modern computational approaches offer an inexpensive and fast assessment of protein allosteric ‘wiring.’ However, the computational approaches only offer hypothetical predictions and still need to be tested experimentally. Experimental validation does not require the structural characterization of the protein; instead, a direct test of the predictions in activity assays demonstrates the functioning of the established allosteric control (Vishweshwaraiah et al., 2021b). Here, we discuss computational and experimental approaches to map allosteric communications, and tools used to control protein function through allosteric regulation.

### Allosteric regulation of protein kinases

**Figure 1.**
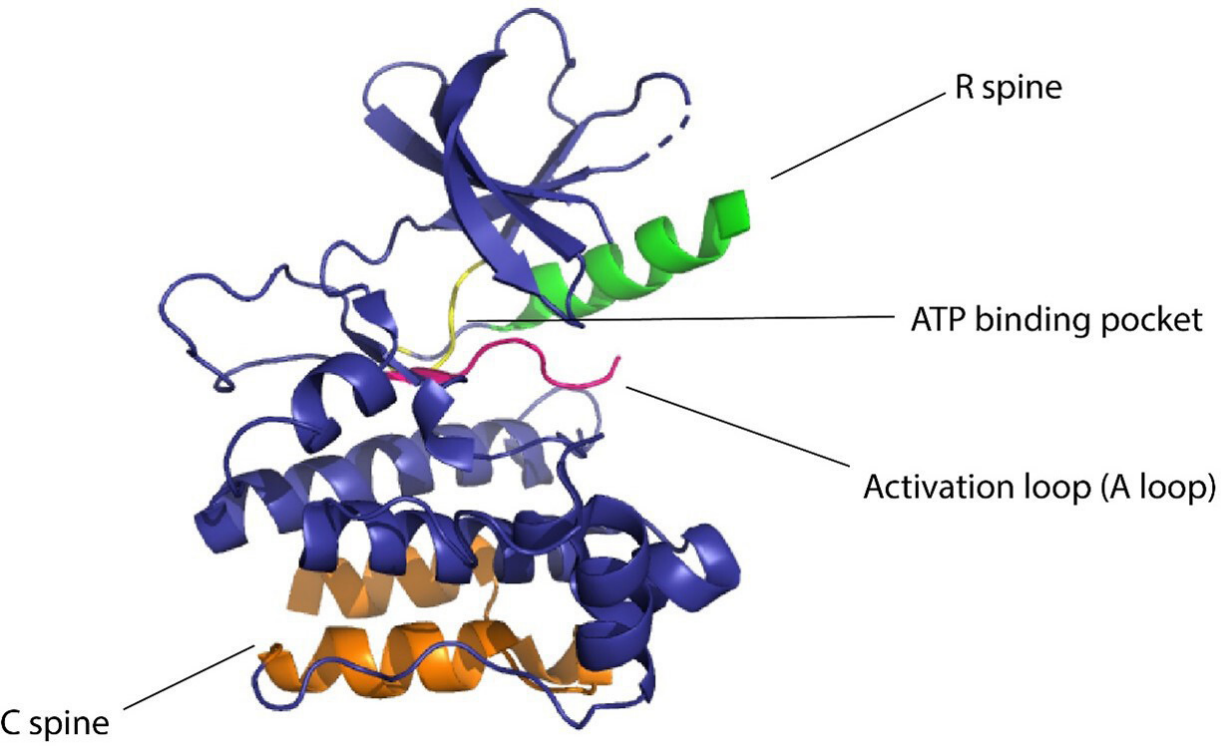
Domains of kinase protein responsible for the regulation of function.

Kinases are considered to be biological switches due to their ability to change structural conformation upon activation (Taylor et al., 2012b). Since kinases make up 2% of the protein-coding genome (Manning et al., 2002) in humans and are implicated in multiple diseases, this family of proteins is an important therapeutic target. Protein kinases phosphorylate serines, threonines, or tyrosines by transferring the γ-phosphate of ATP to the hydroxyl group causing structural changes in the protein, eliciting its function as an enzyme (Cohen, 2002). Kinases have a widespread role in cellular signaling, thereby making them desirable targets for establishing control over cellular processes (Vishweshwaraiah et al., 2021b; Chen et al., 2022b). In eukaryotic cells, across all different types of protein kinases, there are close to 250 residues worthy of conserved sequences (Taylor et al., 2012b). One conserved region is attributed to be the nucleotide-binding domain (NBD), which is a cleft that forms the binding pocket for ATP and is located between the N-terminal lobe and the C-terminal lobe of the protein. The flexibility of the protein is maintained by two ensembles of hydrophobic residues or ‘spines’ that connect the α-helix of the C lobe to the β-sheet of the N lobe (Johnson and Lewis, 2001). These spines are called the ‘regulatory spine (R spine)’ due to their ability to regulate the assembly and disassembly of the active kinase, and the ‘catalytic spine (C spine)’ since these residues balance the catalytic activity of the kinases upon ATP binding. The changes in structure of the kinases due to their complex structure–activity relationship are likely to be resolved due to the presence of the spines (Taylor et al., 2012a). The dynamic structure of kinases has proved to be of great importance for the functionality of the protein and the NMR structures confirming these dynamic structural changes have underscored the presence and importance of allostery of protein kinases (Masterson et al., 2012; Figure 1).

**Figure 2.**
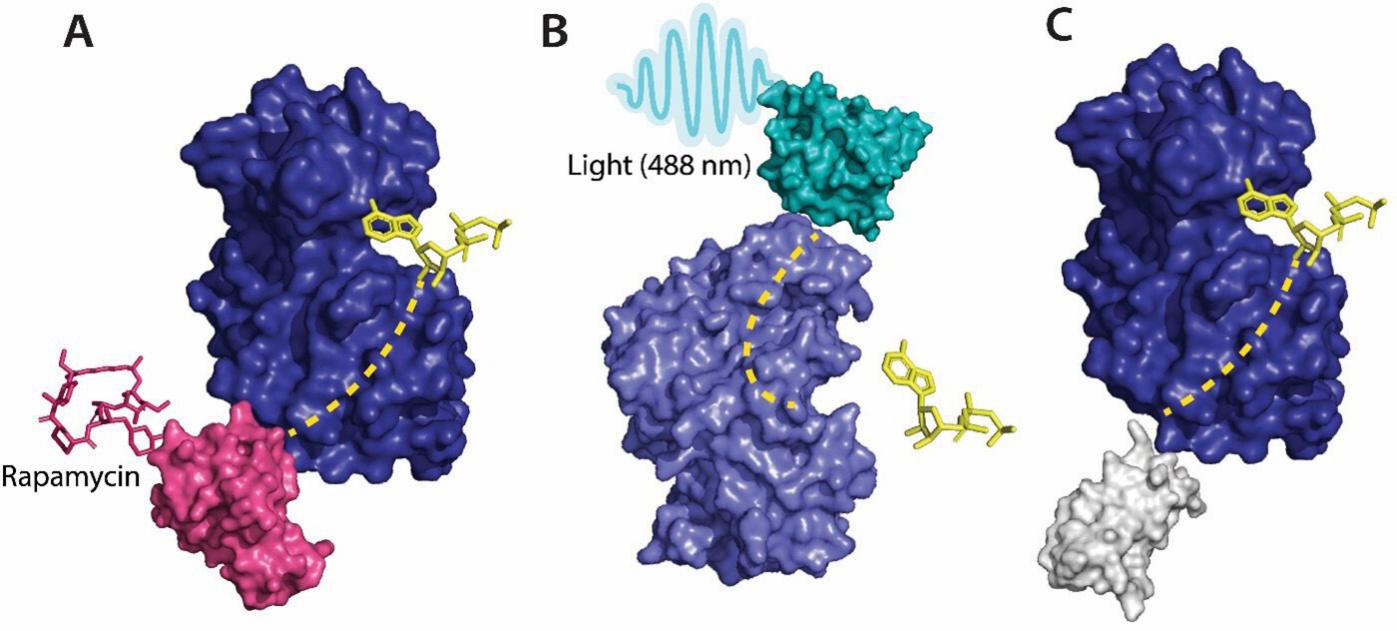
Various tools used as allosteric regulators. (**A**) Allosteric activation of kinase (ERK2 kinase, PDB ID: 4GT3) by uniRapR (PDB ID: 7F2J) domain that binds to small molecule (rapamycin) causing activation of the protein. (**B**) Allosteric inhibition of kinase (ERK2 kinase, PDB ID: 4GT3) by insertion of an optogenetic control protein, LOV2 (PDB ID: 2V0W), causing conformational change upon irradiation by blue light. (**C**) Activation of kinase using monobodies (PDB ID: 3RZW). Figure 2—source data 1.Source data for Figure 2.

Several approaches have been developed using the preexisting tools to control protein function for the allosteric regulation of protein kinases. Kinase activity can be controlled by ligands (Zorba et al., 2019), made use of monobodies (Sha et al., 2017) (a type of synthetic binding protein) to control allostery in Aurora A (AurA) (Zhou et al., 1998). AurA is a protein kinase responsible for the maturation of the centrosome and duplication through its effect on the microtubule spindle formation in mitosis (Glover et al., 1995). AurA serves to be a major oncogene since its overexpression has been associated with a variety of cancers. The authors used monobodies (Koide et al., 2012) to bind to the allosteric sites of AurA and produce complete inhibition or complete activation without having to actively compete with ATP to bind to the NBD. The effect of the binding of monobodies to AurA and the activity of the protein were mapped using X-ray crystallography. The binding of the monobody showed a 20-fold inhibition of the kinases, proving its effectiveness as an allosteric inhibitor and demonstrating promising therapeutic potential. Wojcik et al., 2016 designed high-affinity monobodies to bind to the kinase Bcr-Abl formed by the fusion of breakpoint cluster region (Bcr) and Abelson tyrosine kinase 1 (Abl1). Bcr-Abl activity leads to the development of chronic myelogenous leukemia (Deininger et al., 2000). Studies have shown an interaction between Src homology domain (SH2) and tyrosine kinase, critical to kinase activity and mutations arising at these interfaces (I164E), and leads to inhibition of the kinase activity and oncogenesis induced by Bcr-Abl (Grebien et al., 2011). The authors developed monobodies for these critical residues to control the kinase activity of Bcr-Abl allosterically. The monobodies were developed using combinatorial synthesis and the binding affinity was tested using chemical shift perturbation assays that identify binding sites and predict the binding efficiency of ligands. Inhibition of interaction of the SH2 domain and the kinase domain due to monobodies decreased the activity of Bcr-Abl (validated by western blot and immunohistochemistry experiments). Monobodies can be used as a tool for controlling kinase activity, providing an alternative approach to chemogenetic and optogenetic control of protein function as monobodies do not require engineering of the target kinase (Figure 2).

Dagliyan et al., 2017 developed a rapamycin-binding domain that destabilized the NBD, thereby disrupting the kinase activity in the P21-activated kinase (PAK1). PAK1 is known to play a role in angiogenesis, neurogenesis, and cell motility (Hofmann et al., 2004). The dysregulation of this protein leads to the development of speech impairment, seizures, and macrocephaly. The authors developed a protein domain that is activated upon dopamine binding to the PAK1 domain. PAK1 is overexpressed in breast cancer and hence is identified as an oncogene (Radu et al., 2014). The authors tested this rapamycin-PAK construct (RapR-PAK1) in breast cancer cells to observe changes in the kinase activity due to the effect of rapamycin. The construct was successfully able to control the kinase activity of PAK, demonstrating the applicability of ligands to control protein activity allosterically by either inhibiting activity or inducing it.

Karginov et al., 2011 used a similar strategy for regulating kinase activity, but instead of using a ligand-based approach, the authors used light as the input signal for controlling activity. The authors built a photoactivatable analog of rapamycin to regulate the dimerization of proteins. The photo-inducible small molecules are generated by the installation of ‘caging groups’ (Riggsbee and Deiters, 2010; Mayer and Heckel, 2006) that keep the molecule inactive until removed. Caging groups sequester the site on the ligand necessary for activation. Dissociation of the caging groups from the activation site through irradiation by light leads to activation of the small molecules. The authors used ‘caged-rapamycin (pRap)’ to regulate both the kinase activity and protein dimerization.

Zhou et al., 2017 developed an optically inducible, single-chain protein that could undergo conformational changes upon exposure to light. The authors induced modifications to DronpaN145, a photo-inducible green fluorescent protein to form pdDronpa1 (Zhou et al., 2012) that could dimerize at the kinase active site in the presence of 500 nm cyan and stymie kinase activity. Upon removal of light, the kinase activity was restored. The authors used dual-specificity mitogen-activated protein kinase 1 (MEK1) to test the pdDronpa1 construct. The authors were able to successfully control the kinase activity of MEK1, providing a unique and versatile approach toward optogenetics.

Vishweshwaraiah et al., 2021a developed a protein-based nanocomputing agent (Dokholyan, 2021) containing two inputs that had opposing effects on the kinase activity, forming a logic OR gate to regulate allosteric communications. The authors inserted a chemogenetic (uniRapR; Dagliyan et al., 2013) and an optogenetic domain (LOV2; Lee et al., 2008) within FAK. The uniRapR construct was inserted in the allosteric site of the kinase domain, predicted using Ohm (Wang et al., 2020), such that it could activate the protein upon interaction with rapamycin. This activation pathway of the protein upon interaction with rapamycin was predicted by discrete molecular dynamics (DMD) simulations. The LOV2 construct was inserted in loop 2 of the FERM domain (allosteric site on FERM domain was predicted using Ohm), leading to an inhibition of kinase activity by inactivation of the protein (Lietha et al., 2007). Inactivation of the protein was predicted using DMD simulations in blue light on/off conditions. The authors observed a decrease in kinase activity upon exposure to blue light and an increase in kinase activity upon exposure to rapamycin upon live cell imaging (changes in phenotype were used to determine kinase activity). This successful study provides a basis for the development of rationally designed nano-computing agents and can be used in diagnostics, drug delivery, regulation of cell signaling, and metabolic flux. In order to demonstrate that the nanocomputing agents are effective in other proteins as well, Chen et al. developed an Src-uniRapR-LOV2 construct that provided a dual control on the function of Src kinase. The uniRapR construct was able to activate the Src kinase while the LOV2 construct rendered the protein inactive upon irradiation by blue light. The on/off protein switch was tested by visualization of the focal adhesion. With rapamycin as the first signal, followed by blue light as the second (Chen et al., 2023). Along with computational methods such as multiple sequence alignments (MSA), molecular dynamics (MD), and DMD simulations, molecular docking and in silico modeling (Yuan et al., 2022). paired with mass spectroscopy, have also been used to define protein structures and map allosteric sites in proteins. Through microscopic techniques, such as cryo-EM, two-dimensional structures of proteins can be acquired and morphed into three-dimensional models using artificial intelligence (Chirigati, 2021). In addition to small molecules, biologics such as antibodies or various input stimuli (such as pH, temperature, or pressure) can be made use of to regulate protein function by exploitation of allosteric communication. Protein allostery proves to be an essential tool in controlling protein function in translational research.

### Methods to map allosteric communications

Computer simulations developed to predict allosteric communications offer faster and more cost-effective methods of studying allosteric communication in proteins compared to traditional experimental structural characterization techniques (Dokholyan, 2016). Experimentally, NMR spectroscopy and X-ray crystallography are used in conjunction to map allostery, Boulton and Melacini, 2016 hypothesized that the crystal structures of proteins solved by these techniques would provide information about the changes in the conformation of proteins upon binding of an allosteric regulator. Sites that are susceptible to a conformational change upon binding of the modulator/regulator were termed ‘hot spots.’ The high-resolution images from NMR spectroscopy provided the opportunity to study the conformational dynamics as a result of allostery. By combining the high-resolution images of the protein structure and the MD simulations, predictions can be made about the allosteric communications within proteins. Lisi and Loria, 2017 described allostery using NMR studies paired with the computational predictions in enzyme catalysts; PTP1B (protein tyrosine phosphatase 1B) (Zhang, 2017), which is known to be associated with obesity and diabetes, and PKA-C (protein kinase C) (Xiao et al., 2015), which is responsible for cell signaling and proliferation. Jain et al., 2023 used NMR spectroscopy to configure the active and inactive structures of Cdc42-LOV2 construct. Light oxygen voltage (LOV2) protein is used as an optogenetic tool since it is active under blue light and is used to control protein function allosterically. The authors elucidated the structural changes of the Cdc42-LOV2 occurring upon irradiation by blue light converting the construct from an inactive to an active state. This provides an insight on the allosteric communication within proteins through structural elucidation. NMR spectroscopy is able to map both structural and dynamic changes that occur within an allosteric system, but may not be sufficient to determine the structural and dynamic changes occurring in complex allosteric systems (Boulton and Melacini, 2016).

Computational methods provide a faster means of deciphering protein interactions. Gadiyaram et al., 2019 developed a graph spectral analysis of protein structures to predict allostery using non-covalent interactions between amino acid residues to create a protein structure network. The authors put their theories to the test by predicting the side-chain interactions between residues that form the G-protein coupled receptors (GPCRs) (specifically the β2-adrenergic receptor) and the HIV proteases. Another computational strategy, MSA (Sofi and Masoodi, 2022), is the process of aligning two or more protein sequences to discover the conserved regions of the proteins. Lockless and Ranganathan, 1999 made use of MSA to map allosteric communications in a postsynaptic density protein, *Drosophila* disc large tumor suppressor, and zonula occludens-1 (PDZ) protein domain. They hypothesized that perturbations in one residue of the protein sequence would cause thermodynamic changes in the residue adjacent to it. If the protein sequences are conserved, similar changes in the vibrational frequencies should be seen in all proteins possessing the conserved residue sequence. The theory had two developmental parameters – conservation of the residues at a given site of the protein as observed in the MSA, and statistical coupling of two residues (nodes) of the given amino acid sequence. This method was validated by analyzing the conserved sequences of the PDZ and the Poxvirus and zinc finger (POZ) protein domains using statistical coupling analysis (Teşileanu et al., 2015) and thermodynamic mutation cycles (Pagano et al., 2021), which measures the strength of the interaction of the residues. Ambroggio and Kuhlman, 2006 developed an algorithm to engineer proteins that can undergo structural protein-folding changes using MSA. The algorithm Ambroggio and Kulhman developed could predict stable structural conformations of both the changed and native protein instead of one protein conformation favoring the other. To test the algorithm, the authors made use of the Sw2 sequence, which could switch the conformations between the coiled coil and the zinc finger states. The information provided by MSA is susceptible to errors (Kemena and Notredame, 2009) as the ‘true alignment’ of proteins is not possible limiting the accuracy of allosteric communication predictions using MSA (Nuin et al., 2006).

The calculations necessary to describe protein structure flexibility and allostery of large molecular systems are computationally intensive. MD simulations help to overcome this limitation in predicting the interactions between amino acid residues. MD simulations use atomic interaction approximations to simulate atomic motions on the basis of Newtonian physics and reduce the computational complexity of this systems (Durrant and McCammon, 2011). MD simulations are utilized to develop algorithms that predict allosteric communications. The algorithms not only help in predicting allosteric communication pathways within proteins but also help in determining the potential allosteric sites in the protein. Proctor et al., 2015 analyzed the allosteric communications in mutations of cystic fibrosis transmembrane conductance regulator protein (CFTR) and identified the allosteric hot spots using molecular dynamic simulations. These hot spots could be targeted to restore the transcription and translation of CFTR and provide an allosteric therapeutic approach. MD simulations can predict residue interactions within proteins at a high spatiotemporal resolution and provide a faster method of analysis of allosteric communication within a protein compared to experimental methods used for structure elucidation. The timescales play a role in predicting residue interactions since allosteric communications are dynamic in nature. Short time scales (nanosecond) are not always accurately predicted by MD simulations (Hertig et al., 2016); this can be overcome using coarse-grained simulations (a type of multiscale modeling method that simulates complex behavior of system in a more realistic timescale) or DMD simulations that reach time scales in the range of microseconds (Shirvanyants et al., 2012; Proctor et al., 2011). DMD simulations have been successfully used to predict allosteric communications in proteins (Bowerman and Wereszczynski, 2016; Dokholyan et al., 1998).

Tee et al., 2021 developed a structure-based statistical mechanical model of allostery (SBSMMA). This model predicts allostery by quantifying the thermodynamic perturbations of proteins (upon ligand binding or mutations in the protein) by estimating the free energy of every residue present in the ligand-bound and ligand-free proteins using an allosteric signaling map (ASM). SBSMMA identifies potential exosites, protein regions that are likely to govern the allosteric regulation through reverse perturbations. AlloSigMA (Guarnera et al., 2017) is a server that provides a quantitative assessment of protein-energy dynamics by estimating the changes in the relative energy of the residues at the allosteric site. AlloMAPS (Tan et al., 2019) is a database that provides data on the predicted energy changes and the allosteric pathways of the molecule predicted using SBSMMA (Tee et al., 2021). SBSMMA provides a way to discover allosteric sites on the protein and elucidate allosteric mechanisms within proteins. Wah Tan et al., 2022 developed a protocol for designing allosteric modulators using a fragment-based approach based on the knowledge of allosteric interaction sites on a protein using SBSMMA. Fragment-based approach (Scott et al., 2012) of drug design is the development of an entire small molecule by joining smaller, low molecular-mass fragments that show similar interactions with the target. This method was developed by Jencks, 1981 and was initially used for building small molecules for kinases. Fragment-based approach can be used to map protein–protein interactions, transcription factors, and protein chaperons (Chen et al., 2010).

Treating the allosteric phenomenon as signal propagation through heterogeneous media, Wang et al., 2020 designed a physics-based computational approach for mapping allosteric communications called Ohm (Wang et al., 2020). The multifaceted platform can be used to predict the allosteric sites along with the possible pathways, identify the critical residues, and predict the possible allosteric correlation between the residues. The platform is built on a perturbation propagation algorithm that measures the perturbation of residues by calculating the probability of interactions between the two residues (obtained by a probability matrix). The predictions were validated by predicting the allosteric communication during phosphorylation of CheY (Lee et al., 2001) (a protein found in the flagellar motor of bacteria) and the Caspase-I protein (Clark, 2016) (involved in inflammatory and host-defense response). Allosteric communication within proteins can be predicted by a variety of computational methods that provide closely realistic predictions in a short span of time. These predictions can be validated using experimental techniques.

### Tools to control allostery

Conceptually, a ‘sensor’ domain is engineered into an allosteric site to establish allosteric control over a protein. This sensor domain responds to stimuli, external or internal, and undergoes conformational changes that propagate through allosteric paths to the active site. The active site then is altered upon the application of the stimuli. Some of the examples are where ‘sensor’ proteins interact with small molecules or light and produce a conformational and dynamic change in the target protein leading to either activation or deactivation of the protein.

### Optogenetic control

Photoreceptors are molecules that can change their conformation upon irradiation by light. The light-sensitive domains of the photoreceptors can be used as sensor domains to control protein function (Padmanabhan et al., 2022). The phytochrome B (PhyB) (Ni et al., 1999) is used extensively as a red light photoreceptor since many optical systems do not have far-infrared light range lasers, and red light photoreceptors have limitations to their usage (Ni et al., 1999). Light oxygen voltage (LOV2) and cryptochromes are the two main types of blue light photoreceptors used to control protein function. The (LOV2) domain of phototropin 1 (Halavaty and Moffat, 2007) obtained from *Avena sativa* has been shown to produce dramatic conformational changes upon exposure to blue light and is used to control protein function. Zimmerman et al., 2016 developed LOV2 photoswitches and validated them by inserting them in various protein domains and determining the activity of the protein upon exposure to blue light. Dagliyan et al., 2016 developed a photo-inhibitable Src kinase domain by inserting LOV2 in the L1 loop connecting β-strands of Src kinase causing inhibition of the kinase upon activation of LOV2. However, insertion of the LOV2 domain into the protein without causing structural perturbations in the native protein structure is difficult due to limitations arising from the lack of availability of caging surfaces leading to non-specific activation of the photoreceptors. Caging surfaces or photocages are photosensitive groups that prevent the protein from reacting to small molecules or other proteins that might produce a biological function of the protein. The caging groups ensure activation of proteins only upon irradiation of the proteins by light of suitable wavelength (Bojtár et al., 2020). To overcome this limitation, He et al., 2021 developed a circular permutant of LOV2 (cpLOV2). The cpLOV2 is constructed by introduction of a glycine and serine-rich linker in the surface-exposed loop. The construct remains compatible with the existing LOV2-based engineered proteins with the advantage of having more caging surfaces thus improving its applicability (Figure 2).

**Figure 3.**
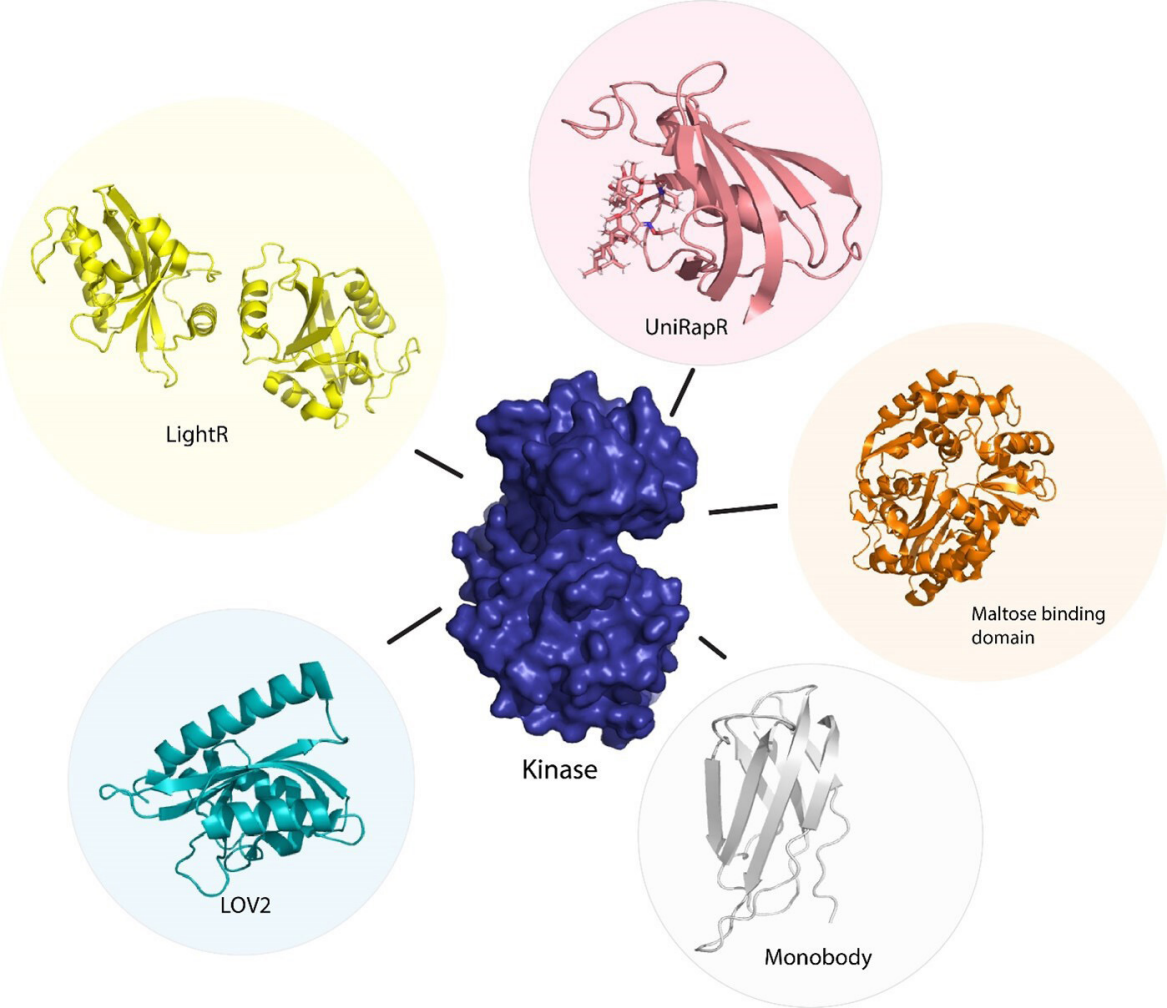
Sensor proteins used to control function of kinase protein allosterically. Chemogenetic tools (LightR, LOV2), optogenetic tool (uniRapR, maltose binding protein), and synthetic protein tool (monobody).

Owing to the difficulty of optogenetic tools to control allosteric activity, arising due to the limitations in achieving subcellular control of activity (Wang et al., 2019, Shaaya et al., 2020) developed an optogenetic tool, LightR, that enables subcellular control of protein and provides a tight temporal regulation of protein activity. LightR comprises of two tandemly connected Vivid photoreceptor (Nihongaki et al., 2014) domains that form an antiparallel homodimer upon irradiation by blue light causing its activation. The use of LightR in controlling allosteric activity was validated in Src kinase, where irradiation with blue light caused activation of Src kinase. LightR provides better spatiotemporal resolution than LOV2 due to the subcellular control of proteins (Figure 3).

### Chemogenetic control

Although optogenetics provides a high resolution of control of protein activity, optogenetic tools are often challenging to translate from cellular to animal models due to the limitation of the extent of absorption of light by animals that are non-transparent. To overcome this limitation, Dagliyan et al., 2013 developed a protein switch (uniRapR) whose activity can be controlled by a small molecule (rapamycin). uniRapR is a complex of FK506-binding protein (FKBP12) and FKBP12-rapamycin binding protein (Choi et al., 1996) built into a single-chain protein. uniRapR binds selectively to rapamycin and upon binding induces a conformational change in the structure. uniRapR can be used to control protein function allosterically. To test the functionality of uniRapR, the authors inserted this construct into Src kinase and focal adhesion kinase (FAK) and compared the changes in phosphorylation of the two enzymes before and after the addition of rapamycin. The activity of uniRapR was further validated using a mutant strain of FAK with a point mutation that caused inhibition of FAK activity; the FAK activity was recovered by uniRapR, which validated its use as an allosteric protein function regulator.

Guntas and Ostermeier, 2004 developed a maltose-binding allosteric regulator using in vitro recombination techniques. The wild-type beta-lactamase enzyme (BLA) activity is rendered unaffected by *Escherichia coli* maltose binding protein. Upon recombination of the two genes, TEM-1-beta-lactamase and *E. coli*. maltose binding gene (Guntas and Ostermeier, 2004), the enzyme activity was seen to be regulated upon the addition of maltose. The change in activity was analyzed by measuring the rate of β-lactam hydrolysis upon an increase in the concentration of maltose. Chemogenetic control of proteins has better in vivo resolution since light can only be absorbed up to a certain depth and has a shorter timescale of activation (Figure 2).

### Conclusion

The human cell kinome consists of 538 kinases that are involved in a variety of cellular processes. Kinases have a conserved active site residue sequence, making it challenging to target a specific kinase with ligands acting on the active site; hence, allosteric regulation of kinases provides the ability to specifically target different types of kinases. Finding the allosteric sites on the proteins and visualizing the changes occurring in the protein conformation is experimentally time-consuming and an expensive endeavor. To overcome this limitation, computational methods are developed that can predict allosteric communication within proteins. Advancements in technology have paved the way to engineer proteins and regulate their activity. Various methods such as chemogenetics, optogenetics, and synthetic proteins are used as allosteric tools to control protein function. Kinases are an important family of proteins as they are implicated in a variety of diseases; hence, allosteric regulation of kinases could be used as a potential therapeutic approach. Combining the various allosteric control tools helps develop nanocomputing agents with more than one input function, leading to a different output function of the protein, providing a means of controlling different aspects of protein function. New tools can be developed that react to different inputs such as temperature, pH, and pressure along with the preexisting tools interacting with light or small molecules. These allosteric control sensor proteins provide a better spatiotemporal resolution in controlling protein function and prove to be a potential therapeutic approach targeting diseases that arise from the dysregulation of proteins.

## Data Availability

The accession codes deposited in PDB for the protein structures used in Figure 2 (4GT3, 2V0W, 3RZW) are included in the manuscript. The following previously published datasets were used: PozharskiE ZhangJ ShapiroP RCSB Protein Data Bank2012ATP-bound form of the ERK2 kinase4GT3 HalavatyAS MoffatK RCSB Protein Data Bank2007N- and C-terminal helices of oat LOV2 (404-546) are involved in light- induced signal transduction (cryo-trapped light structure of LOV2 (404-546))2V0W GilbrethRN KoideS RCSB Protein Data Bank2011Crystal Structure of the Monobody ySMB-9 bound to human SUMO13RZW

## Additional files

MDAR checklist

## References

[bib1] Adams JA (2003). Activation loop phosphorylation and catalysis in protein kinases: is there functional evidence for the autoinhibitor model?. Biochemistry.

[bib2] Ambroggio XI, Kuhlman B (2006). Computational design of a single amino acid sequence that can switch between two distinct protein folds. Journal of the American Chemical Society.

[bib3] Bentea E, Depasquale EAK, O’Donovan SM, Sullivan CR, Simmons M, Meador-Woodruff JH, Zhou Y, Xu C, Bai B, Peng J, Song H, Ming GL, Meller J, Wen Z, McCullumsmith RE (2019). Kinase network dysregulation in a human induced pluripotent stem cell model of DISC1 schizophrenia. Molecular Omics.

[bib4] Bojtár M, Németh K, Domahidy F, Knorr G, Verkman A, Kállay M, Kele P (2020). Conditionally Activatable Visible-Light Photocages. Journal of the American Chemical Society.

[bib5] Boulton S, Melacini G (2016). Advances in NMR Methods To Map Allosteric Sites: From Models to Translation. Chemical Reviews.

[bib6] Bowerman S, Wereszczynski J (2016). Detecting Allosteric Networks Using Molecular Dynamics Simulation. Methods in Enzymology.

[bib7] Chen L, Cressina E, Leeper FJ, Smith AG, Abell C (2010). A fragment-based approach to identifying ligands for riboswitches. ACS Chemical Biology.

[bib8] Chen J, Li Y, Du C, Wei T, Shan T, Wang L (2022a). Protein kinases in cardiovascular diseases. Chinese Medical Journal.

[bib9] Chen J, Vishweshwaraiah YL, Dokholyan NV (2022b). Design and engineering of allosteric communications in proteins. Current Opinion in Structural Biology.

[bib10] Chen J, Vishweshwaraiah YL, Mailman RB, Tabdanov ED, Dokholyan NV (2023). A noncommutative combinatorial protein logic circuit controls cell orientation in nanoenvironments. Science Advances.

[bib11] Cheng HC, Qi RZ, Paudel H, Zhu HJ (2011). Regulation and function of protein kinases and phosphatases. Enzyme Research.

[bib12] Chirigati F (2021). Predicting protein structure from cryo-EM data. Nature Computational Science.

[bib13] Choi J, Chen J, Schreiber SL, Clardy J (1996). Structure of the FKBP12-Rapamycin Complex Interacting with Binding Domain of Human FRAP. Science.

[bib14] Clark AC (2016). Caspase Allostery and Conformational Selection. Chemical Reviews.

[bib15] Cohen P (2002). The origins of protein phosphorylation. Nature Cell Biology.

[bib16] Cui Q, Karplus M (2008). Allostery and cooperativity revisited. Protein Science.

[bib17] Dagliyan O, Shirvanyants D, Karginov AV, Ding F, Fee L, Chandrasekaran SN, Freisinger CM, Smolen GA, Huttenlocher A, Hahn KM, Dokholyan NV (2013). Rational design of a ligand-controlled protein conformational switch. PNAS.

[bib18] Dagliyan O, Tarnawski M, Chu PH, Shirvanyants D, Schlichting I, Dokholyan NV, Hahn KM (2016). Engineering extrinsic disorder to control protein activity in living cells. Science.

[bib19] Dagliyan O, Karginov AV, Yagishita S, Gale ME, Wang H, DerMardirossian C, Wells CM, Dokholyan NV, Kasai H, Hahn KM (2017). Engineering Pak1 Allosteric Switches. ACS Synthetic Biology.

[bib20] Deininger MWN, Goldman JM, Melo JV (2000). The molecular biology of chronic myeloid leukemia. Blood.

[bib21] Dixon RDS, Chen Y, Ding F, Khare SD, Prutzman KC, Schaller MD, Campbell SL, Dokholyan NV (2004). New insights into FAK signaling and localization based on detection of a FAT domain folding intermediate. Structure.

[bib22] Dokholyan NV, Buldyrev SV, Stanley HE, Shakhnovich EI (1998). Discrete molecular dynamics studies of the folding of a protein-like model. Folding & Design.

[bib23] Dokholyan NV (2016). Controlling Allosteric Networks in Proteins. Chemical Reviews.

[bib24] Dokholyan NV (2021). Nanoscale programming of cellular and physiological phenotypes: inorganic meets organic programming. NPJ Systems Biology and Applications.

[bib25] Durrant JD, McCammon JA (2011). Molecular dynamics simulations and drug discovery. BMC Biology.

[bib26] Gadiyaram V, Vishveshwara S, Vishveshwara S (2019). From Quantum Chemistry to Networks in Biology: A Graph Spectral Approach to Protein Structure Analyses. Journal of Chemical Information and Modeling.

[bib27] Glover DM, Leibowitz MH, McLean DA, Parry H (1995). Mutations in aurora prevent centrosome separation leading to the formation of monopolar spindles. Cell.

[bib28] Grebien F, Hantschel O, Wojcik J, Kaupe I, Kovacic B, Wyrzucki AM, Gish GD, Cerny-Reiterer S, Koide A, Beug H, Pawson T, Valent P, Koide S, Superti-Furga G (2011). Targeting the SH2-kinase interface in Bcr-Abl inhibits leukemogenesis. Cell.

[bib29] Grutsch S, Brüschweiler S, Tollinger M (2016). NMR Methods to Study Dynamic Allostery. PLOS Computational Biology.

[bib30] Guarnera E, Tan ZW, Zheng Z, Berezovsky IN (2017). AlloSigMA: allosteric signaling and mutation analysis server. Bioinformatics.

[bib31] Guntas G, Ostermeier M (2004). Creation of an allosteric enzyme by domain insertion. Journal of Molecular Biology.

[bib32] Guo J, Zhou HX (2016). Protein Allostery and Conformational Dynamics. Chemical Reviews.

[bib33] Halavaty AS, Moffat K (2007). N- and C-terminal flanking regions modulate light-induced signal transduction in the LOV2 domain of the blue light sensor phototropin 1 from Avena sativa. Biochemistry.

[bib34] He L, Tan P, Zhu L, Huang K, Nguyen NT, Wang R, Guo L, Li L, Yang Y, Huang Z, Huang Y, Han G, Wang J, Zhou Y (2021). Circularly permuted LOV2 as a modular photoswitch for optogenetic engineering. Nature Chemical Biology.

[bib35] Hertig S, Latorraca NR, Dror RO (2016). Revealing Atomic-Level Mechanisms of Protein Allostery with Molecular Dynamics Simulations. PLOS Computational Biology.

[bib36] Hofmann C, Shepelev M, Chernoff J (2004). The genetics of Pak. Journal of Cell Science.

[bib37] Jain A, Dokholyan NV, Lee AL (2023). Allosteric inactivation of an engineered optogenetic GTPase. PNAS.

[bib38] Jencks WP (1981). On the attribution and additivity of binding energies. PNAS.

[bib39] Johnson LN, Lewis RJ (2001). Structural basis for control by phosphorylation. Chemical Reviews.

[bib40] Karginov AV, Zou Y, Shirvanyants D, Kota P, Dokholyan NV, Young DD, Hahn KM, Deiters A (2011). Light regulation of protein dimerization and kinase activity in living cells using photocaged rapamycin and engineered FKBP. Journal of the American Chemical Society.

[bib41] Keedy DA (2019). Journey to the center of the protein: allostery from multitemperature multiconformer X-ray crystallography. Acta Crystallographica Section D Structural Biology.

[bib42] Kemena C, Notredame C (2009). Upcoming challenges for multiple sequence alignment methods in the high-throughput era. Bioinformatics.

[bib43] Koide A, Wojcik J, Gilbreth RN, Hoey RJ, Koide S (2012). Teaching an old scaffold new tricks: monobodies constructed using alternative surfaces of the FN3 scaffold. Journal of Molecular Biology.

[bib44] Lee SY, Cho H, Pelton JG, Yan D, Berry EA, Wemmer DE (2001). Crystal structure of activated CheY. Journal of Biological Chemistry.

[bib45] Lee J, Natarajan M, Nashine VC, Socolich M, Vo T, Russ WP, Benkovic SJ, Ranganathan R (2008). Surface sites for engineering allosteric control in proteins. Science.

[bib46] Li M, Rehman AU, Liu Y, Chen K, Lu S (2021). Dual roles of ATP-binding site in protein kinases: Orthosteric inhibition and allosteric regulation. Advances in Protein Chemistry and Structural Biology.

[bib47] Lietha D, Cai X, Ceccarelli DFJ, Li Y, Schaller MD, Eck MJ (2007). Structural basis for the autoinhibition of focal adhesion kinase. Cell.

[bib48] Lisi GP, Loria JP (2017). Allostery in enzyme catalysis. Current Opinion in Structural Biology.

[bib49] Lockless SW, Ranganathan R (1999). Evolutionarily conserved pathways of energetic connectivity in protein families. Science.

[bib50] Manning G, Whyte DB, Martinez R, Hunter T, Sudarsanam S (2002). The protein kinase complement of the human genome. Science.

[bib51] Masterson LR, Cembran A, Shi L, Veglia G (2012). Allostery and binding cooperativity of the catalytic subunit of protein kinase A by NMR spectroscopy and molecular dynamics simulations. Advances in Protein Chemistry and Structural Biology.

[bib52] Mayer G, Heckel A (2006). Biologically active molecules with a “light switch". Angewandte Chemie.

[bib53] Ni M, Tepperman JM, Quail PH (1999). Binding of phytochrome B to its nuclear signalling partner PIF3 is reversibly induced by light. Nature.

[bib54] Nihongaki Y, Suzuki H, Kawano F, Sato M (2014). Genetically engineered photoinducible homodimerization system with improved dimer-forming efficiency. ACS Chemical Biology.

[bib55] Nuin PAS, Wang Z, Tillier ERM (2006). The accuracy of several multiple sequence alignment programs for proteins. BMC Bioinformatics.

[bib56] Nussinov R, Tsai CJ (2013). Allostery in disease and in drug discovery. Cell.

[bib57] Ohno M (2014). Roles of eIF2α kinases in the pathogenesis of Alzheimer’s disease. Frontiers in Molecular Neuroscience.

[bib58] Padmanabhan S, Pérez-Castaño R, Osete-Alcaraz L, Polanco MC, Elías-Arnanz M (2022). Vitamin B12 photoreceptors. Vitamins and Hormones.

[bib59] Pagano L, Toto A, Malagrinò F, Visconti L, Jemth P, Gianni S (2021). Double Mutant Cycles as a Tool to Address Folding, Binding, and Allostery. International Journal of Molecular Sciences.

[bib60] Proctor EA, Ding F, Dokholyan NV (2011). Discrete molecular dynamics. WIREs Computational Molecular Science.

[bib61] Proctor EA, Kota P, Aleksandrov AA, He L, Riordan JR, Dokholyan NV (2015). Rational coupled dynamics Network manipulation rescues Disease-Relevant Mutant Cystic Fibrosis transmembrane conductance regulator. Chemical Science.

[bib62] Radu M, Semenova G, Kosoff R, Chernoff J (2014). PAK signalling during the development and progression of cancer. Nature Reviews. Cancer.

[bib63] Riggsbee CW, Deiters A (2010). Recent advances in the photochemical control of protein function. Trends in Biotechnology.

[bib64] Sarkar N, Singh A, Kumar P, Kaushik M (2023). Protein kinases: Role of their dysregulation in carcinogenesis, identification and inhibition. Drug Research.

[bib65] Scott DE, Coyne AG, Hudson SA, Abell C (2012). Fragment-based approaches in drug discovery and chemical biology. Biochemistry.

[bib66] Sha F, Salzman G, Gupta A, Koide S (2017). Monobodies and other synthetic binding proteins for expanding protein science. Protein Science: A Publication of the Protein Society.

[bib67] Shaaya M, Fauser J, Zhurikhina A, Conage-Pough JE, Huyot V, Brennan M, Flower CT, Matsche J, Khan S, Natarajan V, Rehman J, Kota P, White FM, Tsygankov D, Karginov AV (2020). Light-regulated allosteric switch enables temporal and subcellular control of enzyme activity. eLife.

[bib68] Shirvanyants D, Ding F, Tsao D, Ramachandran S, Dokholyan NV (2012). Discrete molecular dynamics: an efficient and versatile simulation method for fine protein characterization. The Journal of Physical Chemistry. B.

[bib69] Soderling TR (1990). Protein kinases. Regulation by autoinhibitory domains. The Journal of Biological Chemistry.

[bib70] Sofi MY, Masoodi KZ, Shafi A (2022). Bioinformatics for Everyone.

[bib71] Tan ZW, Tee WV, Guarnera E, Booth L, Berezovsky IN (2019). AlloMAPS: allosteric mutation analysis and polymorphism of signaling database. Nucleic Acids Research.

[bib72] Taylor SS, Kornev AP (2011). Protein kinases: evolution of dynamic regulatory proteins. Trends in Biochemical Sciences.

[bib73] Taylor SS, Ilouz R, Zhang P, Kornev AP (2012a). Assembly of allosteric macromolecular switches: lessons from PKA. Nature Reviews. Molecular Cell Biology.

[bib74] Taylor SS, Keshwani MM, Steichen JM, Kornev AP (2012b). Evolution of the eukaryotic protein kinases as dynamic molecular switches. Philosophical Transactions of the Royal Society of London. Series B, Biological Sciences.

[bib75] Tee WV, Tan ZW, Lee K, Guarnera E, Berezovsky IN (2021). Exploring the Allosteric Territory of Protein Function. The Journal of Physical Chemistry. B.

[bib76] Teşileanu T, Colwell LJ, Leibler S (2015). Protein sectors: statistical coupling analysis versus conservation. PLOS Computational Biology.

[bib77] Vishweshwaraiah YL, Chen J, Chirasani VR, Tabdanov ED, Dokholyan NV (2021a). Two-input protein logic gate for computation in living cells. Nature Communications.

[bib78] Vishweshwaraiah YL, Chen J, Dokholyan NV (2021b). Engineering an Allosteric Control of Protein Function. The Journal of Physical Chemistry. B.

[bib79] Wah Tan Z, Tee WV, Berezovsky IN (2022). Learning About Allosteric Drugs and Ways to Design Them. Journal of Molecular Biology.

[bib80] Wang J, Liu Y, Liu Y, Zheng S, Wang X, Zhao J, Yang F, Zhang G, Wang C, Chen PR (2019). Time-resolved protein activation by proximal decaging in living systems. Nature.

[bib81] Wang J, Jain A, McDonald LR, Gambogi C, Lee AL, Dokholyan NV (2020). Mapping allosteric communications within individual proteins. Nature Communications.

[bib82] Wojcik J, Lamontanara AJ, Grabe G, Koide A, Akin L, Gerig B, Hantschel O, Koide S (2016). Allosteric inhibition of Bcr-Abl Kinase by high affinity Monobody inhibitors directed to the Src Homology 2 (SH2)-Kinase interface. The Journal of Biological Chemistry.

[bib83] Xiao Y, Liddle JC, Pardi A, Ahn NG (2015). Dynamics of protein kinases: insights from nuclear magnetic resonance. Accounts of Chemical Research.

[bib84] Yuan J, Jiang C, Wang J, Chen CJ, Hao Y, Zhao G, Feng Z, Xie XQ (2022). In Silico prediction and validation of CB2 allosteric binding sites to aid the design of allosteric modulators. Molecules.

[bib85] Zhang ZY (2017). Drugging the undruggable: Therapeutic Potential of Targeting Protein Tyrosine Phosphatases. Accounts of Chemical Research.

[bib86] Zhou H, Kuang J, Zhong L, Kuo WL, Gray JW, Sahin A, Brinkley BR, Sen S (1998). Tumour amplified kinase STK15/BTAK induces centrosome amplification, aneuploidy and transformation. Nature Genetics.

[bib87] Zhou XX, Chung HK, Lam AJ, Lin MZ (2012). Optical control of protein activity by fluorescent protein domains. Science.

[bib88] Zhou XX, Fan LZ, Li P, Shen K, Lin MZ (2017). Optical control of cell signaling by single-chain photoswitchable kinases. Science.

[bib89] Zimmerman SP, Kuhlman B, Yumerefendi H (2016). Engineering and application of LOV2-based photoswitches. Methods in Enzymology.

[bib90] Zorba A, Nguyen V, Koide A, Hoemberger M, Zheng Y, Kutter S, Kim C, Koide S, Kern D (2019). Allosteric modulation of a human protein kinase with monobodies. PNAS.

