# Supplementary material for: Allosteric regulation of kinase activity in living cells
Source: eLife. 2023 Nov 9;12:RP90574. doi: 10.7554/eLife.90574 (PMC10635643; doi:10.7554/eLife.90574)
Supplement: Figure 2—source data 1. [file elife-90574-fig2-data1.docx]

**SOURCE DATA FILE**

**Source data file for protein structures in Figure 2**

**Figure 2** A Allosteric activation of kinase (ERK2 kinase, PDB ID: 4GT3) by uniRapR (PDB ID: 7F2J) domain that binds to small molecule (rapamycin) causing activation of the protein. B Allosteric inhibition of kinase (ERK2 kinase, PDB ID: 4GT3) by insertion of an optogenetic control protein, LOV2 (PDB ID: 2V0W), causing conformational change upon irradiation by blue light. C Activation of kinase using monobodies (PDB ID: 3RZW).

| **Sr. No** | **Protein Name** | **PDB accession code** |
| --- | --- | --- |
| 1. | ERK2 kinase | 4GT3 |
| 2. | uniRapR | 7F2J |
| 3. | LOV2 | 2V0W |
| 4. | Monobodies | 3RZW |
